# Assessment of the differences in Mean Age at Menarche (MAM) among adolescent girls in rural and urban Nigeria: a systematic review

**DOI:** 10.1186/s12889-024-21054-y

**Published:** 2024-12-18

**Authors:** Hilary I. Okagbue, Olugbemisola W. Samuel, Emmanuella C. Nzeribe, Sunday E. Nto, Olukayode E. Dahunsi, Muhammad B. Isa, John Etim, Evelyn E. Orya, Sidney Sampson, Alexey V. Yumashev

**Affiliations:** 1 Sydani Institute for Research and Innovation, Sydani Group, Abuja, Nigeria; 2 Sydani Group, Abuja, Nigeria; 3 Sechenov First Moscow State Medical University, Moscow, Russia

**Keywords:** Correlation, Incidence, Menarche, Menstruation, PRISMA, Review, Statistics

## Abstract

**Introduction:**

Globally, there has been a decline in the age of menarche; the decline is higher in poorer countries than in richer ones. The measurement of the decline was based on the reported mean age at menarche (MAM) across the countries. There is a significant knowledge gap in investigating the generational decline in MAM in low- and median-income countries (LMC). In Nigeria, different studies have reported MAM, but none have attempted to investigate the generational shift in the reported MAM in girls residing in rural and urban areas. This review sought to understand if there is a rural-urban disparity in the MAM.

**Methods:**

Documents were searched in the relevant bibliometric database and Population intervention, comparison(s) and outcome (PICO) framework were used as eligibility criteria for extracting data from the documents based on some inclusion and exclusion criteria. The population are adolescent schoolgirls in rural and urban settlements in Nigeria. The comparator is the age of menarche of urban versus rural adolescent schoolgirls in Nigeria, while the mean age at menarche (MAM) is the outcome. Data quality assessment was done to critically appraise the included studies and enhance. Data were synthesized using narrative review, descriptive and inferential statistics.

**Results:**

Ten articles were included in the study, following the PRISMA framework. The overall mean evaluation of the risk of bias in the individual studies included in the review was computed to be 88%. Generally, there seems to be a decline in the age at menarche from 1976 to 2023. The rural MAM is higher than the urban MAM, and the gap between the two appears to be narrowing. The t-test showed no statistically significant mean differences between the rural and urban mean age at menarche (T = 2.1009, p value = 0.4679). The mean menarcheal age for girls in rural and urban areas is 13.44 and 13.04, respectively. There is a strong positive correlation between the rural and urban MAMs (Pearson = 0.93, *p* < 0.001). The Gaussian kernel estimated a bimodal distribution for rural girls, where they are most likely to experience menarche at 11 and 13 years, respectively, while urban girls are most likely to experience menarche at 13 years. In both locations, the incidence of menarche decreases just after the peak at 13 years.

**Conclusion:**

Although rural girls have delayed menarche, there is no statistically significant mean difference between the age at menarche reported for rural and urban areas in Nigeria. Interventions in the form of counseling and reproductive education are recommended. The review provides a strong foundation for further research and policy development aimed at improving the health and well-being of adolescent girls in Nigeria and other similar settings.

**PROSPERO Registration:**

CRD42024529497

**Supplementary Information:**

The online version contains supplementary material available at 10.1186/s12889-024-21054-y.

## Introduction

Menarche is known as the onset of menstruation and comes with physical, mental, and physiological changes [1]. Etymologically, menarche is derived from two Greek words “*men*” and “*arche*”, where “*men*” denotes moon and ‘a*rche*’ means beginning. The age of menarche heralds the onset of menstruation [2]. This is an important time in every girlchild’s life as it signifies the gradual transition from childhood to adulthood. It is considered the central event of female puberty, and it heralds the era of possibility of fertility [3]. Researchers have found that menarche occurs between two to three years after breast development (thelarche), although this timing between the menarche and thelarche could be elongated or shorten based on genetics, nutrition, overall health, and environmental factors [4].

Understanding the age of menarche in any context is important for patient education and can guide the clinical evaluation of patients to identify any deviation from normal expectations [5].

This is because early menarche is associated with a higher risk of obesity [6], which is associated with various health problems such as diabetes [7] and cardiovascular disease [8], and risk of breast cancer [9] later in life. On the other hand, late menarche may be associated with reduced bone mineral density due to a shorter duration of exposure to estrogen during adolescence, which could potentially increase the risk of osteoporosis and fractures later in life [10]. Furthermore, delayed menarche can be a sign of infertility and other negative health outcomes [11].

Globally, there has been a decline in the age of menarche, with the decline being more pronounced in poorer countries than in richer ones [12]. The measurement of this decline was based on the reported mean age at menarche (MAM) across the countries. A decline from 14.66 years to 12.86 years for the 1932 and 2002 cohort studies has been reported [12]. The decline could be due to changes in socioeconomic status [12], nutritional status [13], genetics [14], advances in healthcare, environmental factors [15], increased body mass index (BMI) [16], lower levels of physical activity, and higher sedentary behavior in children [17], among other factors.

In Nigeria, different studies have reported MAM, but none have attempted to investigate the generational shift in the reported MAM in girls residing in rural and urban areas. Available studies report cross-sectional differences between MAM as summarized in [18]. Hence this paper reports a systematic review of MAM from the available studies that reported MAM for both rural and urban settings in Nigeria. To synthesize the knowledge on this subject and improve health outcomes, this review sought to understand if there exists a rural-urban disparity in the MAM. Examining the differences in the age of menarche between rural and urban areas in Nigeria can provide insights into health disparities. Factors such as access to healthcare, nutrition, education, and environmental conditions can differ between rural and urban settings [19–23], which can influence the age of menarche and overall health outcomes. Understanding these health disparities is crucial as the onset age of menarche is linked to various long-term health implications, such as the likelihood of obesity, diabetes, cardiovascular diseases, breast cancer, osteoporosis, and infertility [6–11]. Identifying rural-urban differences in MAM can inform targeted interventions to address specific needs in these communities, ultimately contributing to improved health outcomes for girls and women in Nigeria.

## Methods

This was a review of publicly available studies conducted as part of a series on understanding gender-based barriers to health outcomes in Nigeria. Methods have been described in detail in previous studies [24, 25]. No ethical approval was required. This study was conducted in line with the PRISMA (Preferred Reporting Items for Systematic Reviews and Meta-Analyses) guidelines [26].

### Hypothesis

There is no statistically significant mean difference between the age at menarche reported for rural and urban areas in Nigeria.

### Eligibility criteria

Population, Intervention, Comparison(s), and Outcome (PICO) are typically used for systematic reviews and meta-analyses of clinical trial studies. For studies without intervention, it is sufficient to use P (Population), C (Comparator) and O (Outcome) only to formulate a research question [27]. The details of the PCO are presented in Table 1.

**Table 1:** PCO framework of the rural versus urban MAM of girls in Nigeria

PCO	Definition
Population	Adolescent schoolgirls in rural and urban settlements in Nigeria.
Comparator	Age of Menarche of urban versus rural adolescent schoolgirls in Nigeria
Outcome	Mean Age at Menarche

### Search strategy

The terms used in the search were identified using the PCO framework. The search strategy included synonyms, Boolean operators, truncations, and limiters. Google Scholar, Scopus, and PubMed data repositories were used to search between February 1 and 14, 2024 for relevant articles on the subject using the keywords ‘Menarche and Nigeria’. These keywords were used in libraries and research repositories and combined using OR and AND - ‘Menarche OR “Menarcheal age” OR “First menstrual flow” AND Nigeria; “Menarche AND Nigeria"’.

The search was further streamlined into “rural” AND “urban”.

The inclusion and exclusion criteria were as follows:

- Cross-sectional studies that reported the mean age at menarche (MAM) for rural and urban settings in Nigeria were included.
- Articles published between 1970 and 2023 were included.
- Qualitative studies and conference papers were excluded.
- Studies that comprised of populations adolescents of which some are schoolgirls were included.
- Cross-sectional studies of post-menopausal women reporting both age at menarche (AAM) and age at menopause were excluded. This exclusion was done to reduce the risk of recall bias [28].
- Studies that did not report MAM were excluded.
- Duplicates from different bibliometric databases were harmonized.
- Only articles written in English were included.

### Identification and selection of studies

The authors followed a predefined set of criteria, as stated earlier, to assess articles for inclusion in the review. All authors agreed on the final selection. Titles and abstracts were screened first. Full texts were consulted where the mean age at menarche (MAM) was not reported in the abstract.

### Quality assessment

Ensuring the integrity and reliability of the data is paramount in systematic reviews. A rigorous data quality assessment was conducted to evaluate the credibility and trustworthiness of the included studies. The quality assessment was done by two authors who independently reviewed articles following the quality assessment questions. Articles were scored one if they met the criterion, and zero otherwise. However, this systematic review included all publications, regardless of quality, to avoid publication bias. Two of the authors assessed the quality and resolved conflicts by defining criteria, comparing scores, and discussing apparent differences. Furthermore, conflicts between the two assessors were handled by involving the third author to break the tie. These were done manually without a software.

This assessment encompassed the examination of various aspects, such as the study design, data collection methods, and sample size. We also assessed the quality of reporting, the risk of bias, and the representativeness of the study populations. This comprehensive data quality assessment enabled us to critically appraise the included studies and enhance the validity and robustness of our systematic review findings. This review adopted the quality assessment questions (checklist) as used in [29]. Questions considered are listed:

i. Were the criteria for inclusion in the sample clearly defined?
ii. Were the study subjects and the setting described in detail?
iii. Were objective, standard criteria used for measurement of the condition?
iv. Were the outcomes validly and reliably measured?
v. Was appropriate statistical analysis used?

### Data extraction and synthesis

Data extraction of relevant findings was conducted using pretested data extraction forms prepared on Microsoft Excel (Microsoft Corporation, Washington, USA). The data extracted included:

The detailed bibliometric of the articles.

- The study periods.
- Reported MAM.
- Reported MAM for urban setting.
- Reported MAM for rural setting.
- Sample size.
- Nature of sample: Adolescents, schoolgirls.
- Study setting: Geopolitical region (GPR)—North-East (NE), North Central (NC), North-West (NW), South-East (SE), South-South (SS), and South-West (SW), all.
- Study design: Cross-sectional, population cross-sectional.
- Study Summary.
- Significance between rural and urban MAM: Yes, no, not reported.

### Data analysis

Data were synthesized using descriptive and inferential statistics, which are important tools in the process of data synthesis and interpretation. Descriptive statistics were used to summarize and describe the characteristics of the included studies, such as sample size, mean, median, range, and standard deviation of the outcome variables. These statistics helped provide an overview of the study populations.

Inferential statistics were used to draw broader conclusions about the rural versus urban reported mean age at menarche (MAM) based on the available evidence extracted from the ten articles. Specifically, t-tests and analysis of variance (ANOVA) were used. A p-value less than or equal to 0.05 was considered significant. Lastly, Kernel density estimation (KDE) was applied to estimate the underlying distribution of MAM between rural and urban areas.

Statistical Product and Service Solutions (SPSS) version 27 was used for all the analyses, except for kernel density estimation, which was done using R software version 4.2.

## Results

### Search results

**Fig. 1:**
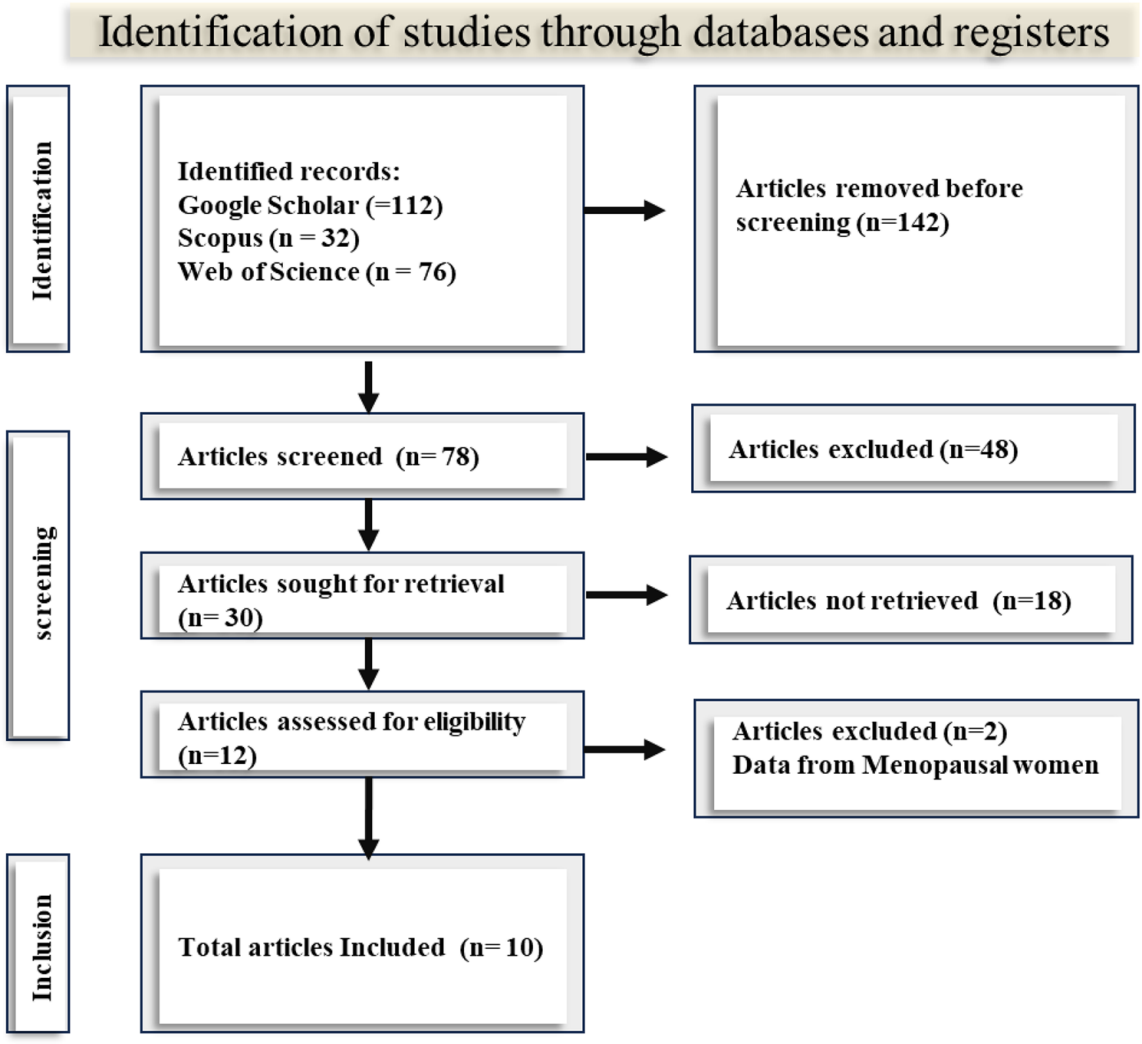
PRISMA flow chart

A total of 220 records were retrieved from the databases – 112 studies in Google Scholar, 32 in Scopus, and 76 in PubMed. The PRISMA flowchart that showed the detailed processes of identification, screening and inclusion that resulted in the final 10 studies retained for synthesis is shown in Fig. 1. The details of the data extraction and synthesis that contained all the variables can be obtained in Supplementary Table S1.

### Quality assessment

The result of the risk assessment is presented in Supplementary Table S2, where 7 out of 10 studies received the maximum score of 100, while the remaining 3 studies were rated at 60%. The overall mean evaluation of the risk of bias in the individual studies included in the review was computed to be 88%. Additionally, the overall median, mode, skewness, and kurtosis were 100, 100, -1.04, and − 1.22, respectively. Negative skewness implies that most of the data and the median are above the mean, while negative kurtosis can indicate that the data points are less clustered around the mean and the presence of a few outliers.

### Overview of included results

The ten articles [30–38], and [39] reported MAM for both rural and urban adolescents and schoolgirls (Supplementary Table S1). The first study was published in 1976 [30], while the last study was published in 2023 [39]. Only 3 studies reported the study period, i.e., when the study was conducted [31, 33, 37]. 60% reported the mean age at menarche (MAM) along with the rural and urban MAM [31, 33, 36–39]. The total sample size was 8585, with a minimum of 243 and a maximum of 2357 participants. 80% reported that their samples were comprised of schoolgirls [31–35, 37–39], while 20% reported that their samples were adolescent girls [30, 36].

Regarding the study area, 70% were from the southern region [30, 32, 34, 35, 37–39], 20% from the northern region [31, 33], and 10% took samples from both regions [36]. The study that took samples from both regions adopted a population cross-sectional design, while the remaining 9 studies used a cross-sectional study design.

Half of the studies concluded that the socioeconomic status of the girls affects the age at menarche [30, 32, 33, 37, 39]. The summary of the remaining 5 studies is as follows: ethnic differences in the age at menarche, sociodemographic factors, prevalence of menstrual problems or disorders, and differences in knowledge of different aspects of menstruation.

Lastly, four studies did not report whether there is a significant mean difference between the age at menarche in rural and urban areas [30, 31, 35, 38], three reported that significant mean differences exist [32, 34, 39], and three reported the absence of such observation [33, 36, 37]. The MAM of rural areas was higher than the urban in all three (3) studies that reported that there are statistically significant mean MAM differences between the rural and urban areas.

### Declining age of menarche

**Fig. 2:**
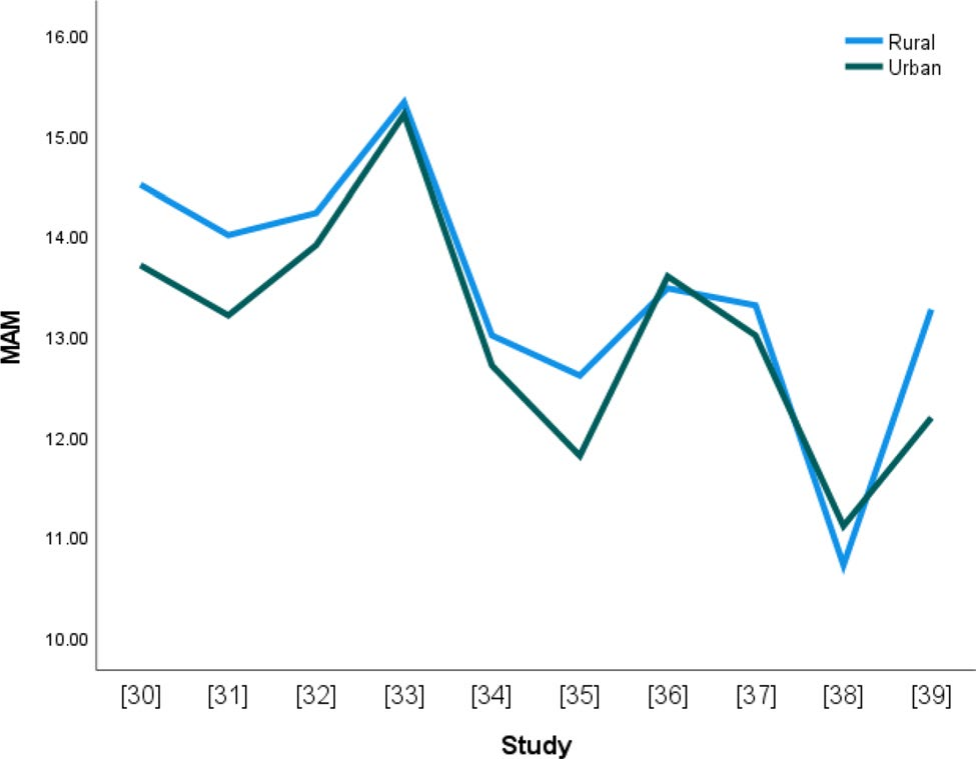
The line graph showing rural and urban MAM

Figure 2 displays the reported mean age at menarche (MAM) for rural and urban areas from the ten studies. Generally, there seems to be a decline in the age at menarche from 1976 to 2023. The rural MAM is higher than the urban MAM, and the gap between the two appears to be narrowing.

### Statistical differences between rural and urban MAM

Statistical tests were conducted to determine the different inferences that could be made about the population of girls living in rural and urban areas in Nigeria.

The mean menarcheal ages for girls in rural and urban areas are 13.44 and 13.04, respectively. Alternatively, on average, girls in rural areas experience menarche at 13 years and 5 months, while urban girls experience it at 13 years. To capture the range of possible values, the 95% confidence intervals of the mean age at menarche are given as follows: the rural mean age at menarche is 13.44 ± 0.39, while the urban mean age at menarche is 13.04 ± 0.37. The t-test showed no statistically significant mean differences between the rural and urban mean age at menarche (T = 2.1009, p-value = 0.4679).

As displayed in Table 2, there are no statistically significant mean menarcheal age differences among the studies that reported overall mean age at menarche for rural and urban girls compared to those that did not report it. Studies that did not report overall mean age at menarche had a mean menarcheal age of 12.85 for girls in urban areas and 13.58 for girls in rural areas. On the other hand, studies that reported overall mean age at menarche had a mean menarcheal age of 13.34 for girls in rural areas and 13.05 for girls in urban areas.

**Table 2:** Test summary of reporting overall MAM

Location	Reported overall MAM	*n*	Mean	F	*P*-value
Rural	Not reported	4	13.58	0.2828	0.8371
	Reported	6	13.34		
Urban	Not reported	4	12.85		
	Reported	6	13.05		

The data were analyzed based on the studies that comprised 1000 or more samples and those with less than 1000 samples. The rationale behind this grouping is because samples above 1000 are more likely to be representative of the broader population, capturing a wider range of individual characteristics and variations, ensuring that the findings are more generalizable. In contrast, samples below 1000 might represent more specific or localized groups, providing valuable insights, but with less generalizability. By comparing the MAM of these groups, the study can assess whether the observed MAM differences hold true across different sample sizes. No statistically mean differences were found between the two as presented in Table 3.

The studies that comprised 1000 and above samples had a lower mean age at menarche for both rural and urban areas than those with samples less than 1000.

**Table 3:** Test summary of sample size

Location	Sample size	*n*	Mean	F	*P*-value
Rural	Less than 1000	7	13.67	0.5540	0.6528
	1000 and above	3	12.89		
Urban	Less than 1000	7	13.04		
	1000 and above	3	12.80		

There are no statistically significant differences in mean age at menarche among the studies that comprised adolescents compared to those comprised of schoolgirls (Table 4). The studies that comprised adolescents had a higher mean age at menarche for both rural and urban areas than those comprised of schoolgirls.

**Table 4:** Test summary of sample composition

Location	Sample composition	*n*	Mean	F	*P*-value
Rural	Adolescent	2	13.99	0.6801	0.5769
	Schoolgirls	8	13.30		
Urban	Adolescent	2	13.65		
	Schoolgirls	8	12.80		

There are no statistically significant differences in mean age at menarche among the studies reported for the northern and southern regions of Nigeria (Table 5). The studies that comprised samples from Northern region had a higher mean age at menarche for both rural and urban areas than those from southern Nigeria.

**Table 5:** Test summary of regional differences

Location	Region	*n*	Mean	F	*P*-value
Rural	North	2	14.66	2.6358	0.0905
	South	7	13.08		
Urban	North	2	14.20		
	South	7	12.52		

There are no statistically significant mean differences in age at menarche among the studies that concluded that socioeconomic or other factors affect menarche (Table 6). The studies that concluded that socioeconomic factors affect menarche had a higher mean age at menarche for both rural and urban areas than those that reported other factors such as sociodemographic factors, menstrual hygiene, and so on.

**Table 6:** Test summary of differences in study summary

Location	Study summary	*n*	Mean	F	*P*-value
Rural	Socioeconomic	5	14.12	2.34764	0.111194
	Others	5	12.76		
Urban	Socioeconomic	5	13.45		
	Others	5	12.48		

Again, as displayed in Table 7, there are no statistically significant mean differences in age at menarche across the studies that did not report or respond yes or no to the question of whether there is a statistical mean age at menarche difference between rural and urban areas (BRU). The studies that concluded that there are no statistically significant mean differences between the age at menarche in rural and urban areas reported a higher mean age at menarche than those that concluded that statistically significant mean differences exist and those that did not report any statistical differences.

**Table 7:** Test summary of studies that reported significance differences

Location	BRU	*n*	Mean	F	*P*-value
Rural	No	3	14.03	1.0361	0.4345
	Not reported	4	12.95		
	Yes	3	13.49		
Urban	No	3	13.93		
	Not reported	4	12.45		
	Yes	3	12.97		

### Correlation between rural and urban MAM

**Fig. 3:**
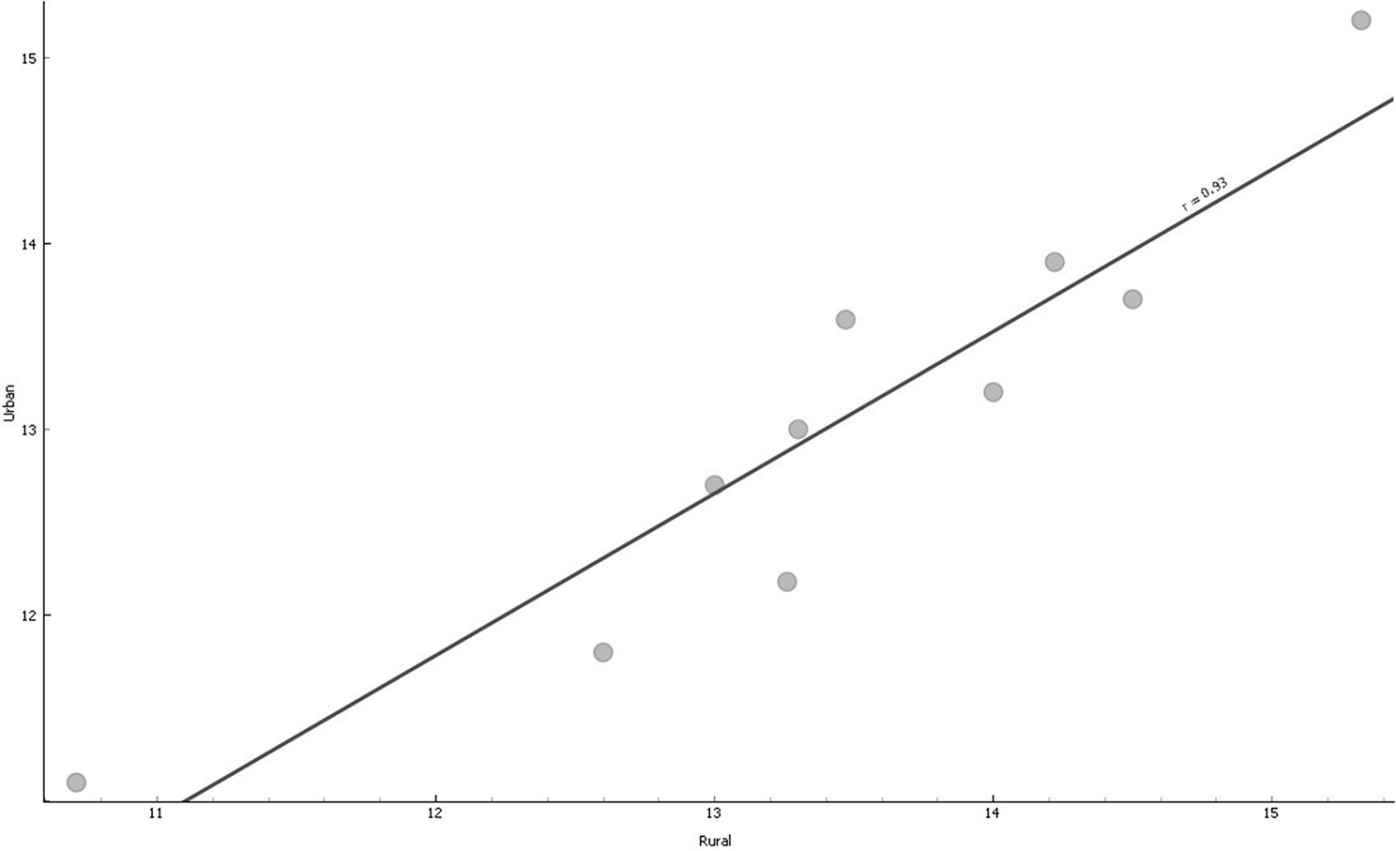
Scatter dots and trend line of Urban Versus Rural MAM

A scatter plot between the rural and urban MAMs depicts a positive correlation. A regression line yielded a strong positive correlation of 0.93 (*p* < 0.001), shown graphically in Fig. 3.

The positive correlation corroborated the line graph that showed that both rural and urban MAMs are declining. It means that MAM in rural and urban settings could be increasing or decreasing simultaneously.

### Kernel Density Estimation

**Fig. 4:**
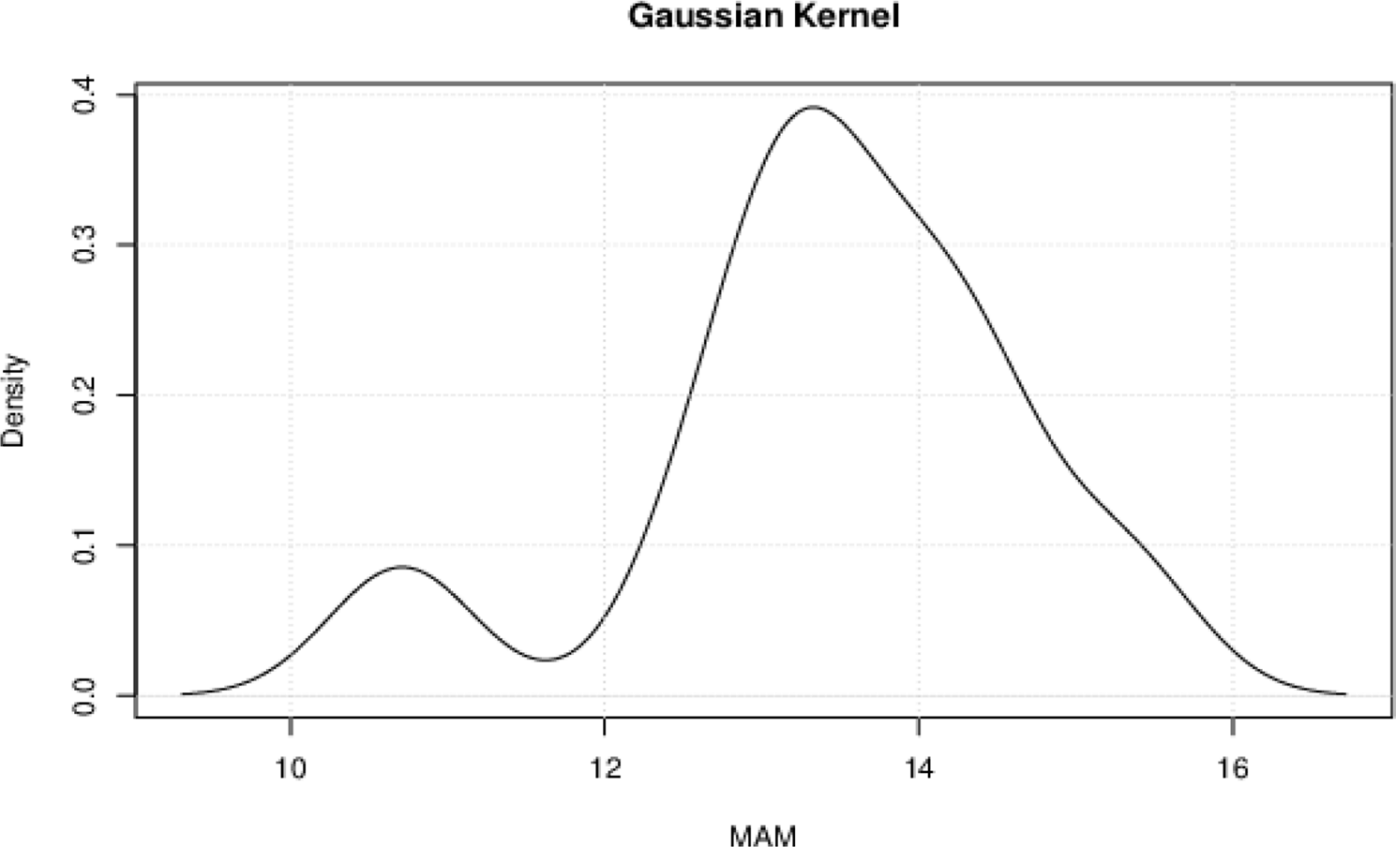
The Gaussian Kernel Density estimation of the probability density of rural MAM

**Fig. 5:**
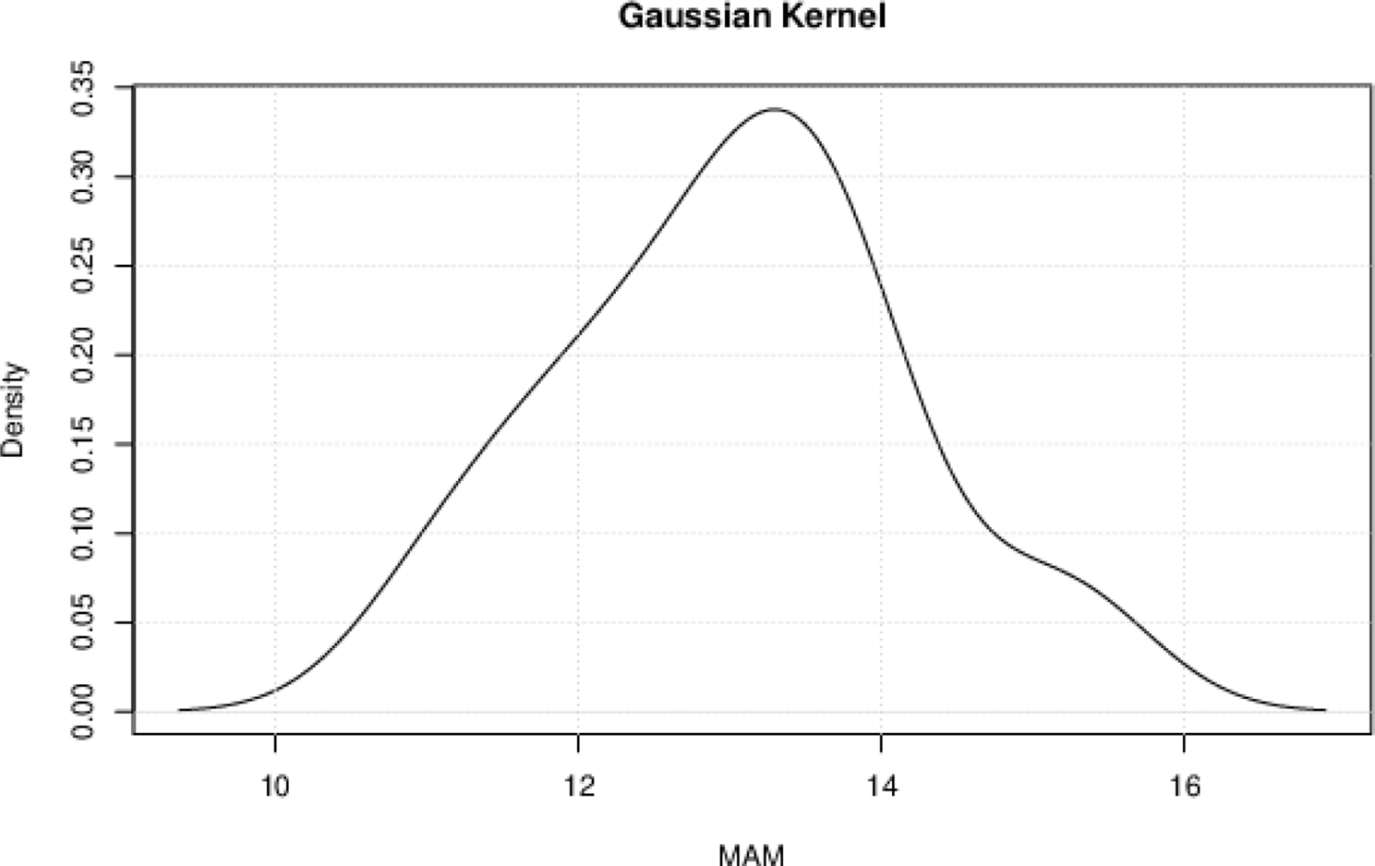
The Gaussian kernel density estimation of the probability density of urban MAM

Kernel density estimation (KDE) is a non-parametric method used to estimate the probability density function of a random variable. In this research, KDE is used to estimate the probability densities of the rural and urban mean age at menarche (MAM) without assuming a specific parametric form. The Gaussian kernel function was used, and plots were obtained for the rural (Fig. 4) and urban (Fig. 5) settlements. The Gaussian kernel is effective in capturing the shape of the underlying distribution and is one of the most widely used kernels in KDE, making it easier to compare results across studies and datasets [40].

The Gaussian kernel estimated a bimodal distribution for rural girls, where they are most likely to experience menarche at 11 and 13 years, respectively. Urban girls are most likely to experience menarche at 13 years. In both locations, the incidence of menarche decreases just after the peaks at 13 years.

## Discussion

### Declining age of menarche in Nigeria

This paper corroborates the earlier study [41] that menarcheal age is declining in Nigeria. The declining menarcheal age is in line with research results in several countries such as Indonesia [42], China [43], South Africa [44], India [45], the United States [46], Italy [47], and Israel [48] that reported declining menarcheal age over the years. However, each study considered different periods, rates of decline, and methodologies that birthed the respective research. Also, this study is equally in line with the study whose submission was that menarcheal age is declining steadily in sub-Saharan Africa [49], and the decline appears to be faster when compared to Europe and other regions of Africa. Factors such as socioeconomic and nutritional status among others have been cited as drivers for the decline [50, 51].

### Rural and urban differences in menarcheal age in Nigeria

This review has provided evidence of no statistically significant mean differences between the rural and urban mean age at menarche, which corroborates the findings of 3 out of the 10 articles systematically reviewed in this paper. Furthermore, on average, girls in rural areas experience menarche later than those in urban areas. This phenomenon could be due to various factors. In rural areas, girls may experience different living conditions and lifestyle factors that can delay the onset of puberty, while those in urban areas have higher nutritional and socioeconomic status that could trigger early onset of menarche. Other factors that could cause disparities in the attainment of menarche include levels of physical activity and exposure to environmental pollutants [52].

Additionally, cultural and socioeconomic factors prevalent in rural settings may also play a role. Understanding these differences can help inform public health interventions and support tailored healthcare strategies for girls in both rural and urban areas. A comparison was done with 17 randomly selected studies with a similar theme (Table 8), and this study corroborated 16 out of the 17 studies. The comparison also found that the difference between rural and urban MAM is generational across different countries, the study design notwithstanding. A further systematic review is recommended to understand the details and the extent and causes of the differences between menarcheal ages in rural and urban settings globally.

**Table 8:** Comparison with studies with similar central theme

Reference	Country	Year published	Study design	Rural > Urban	Urban > Rural
This paper	Nigeria		Systematic Review	Yes	
Łaska-Mierzejewska et al. [53]	Poland	1982	Cohort	Yes	
Ajong et al. [54]	Cameroon	2020	Cross-sectional	Yes	
Choudhary et al. [55]	India	2016	Cross-sectional		Yes
Ohsawa et al. [56]	China	1997	Cross-sectional	Yes	
Said-Mohamed et al. [57]	South Africa	2018	Cross-sectional	Yes	
Zegeye et al. [58]	Ethiopia	2009	Cross-sectional	Yes	
Mokha et al. [59]	India	2006	Cross-sectional	Yes	
Song et al. [60]	China	2014	Cross-sectional	Yes	
Marván et al. [61]	Mexico	2020	Health Survey	Yes	
Zakaria et al. [62]	Morocco	2019	Cross-sectional	Yes	
Odongkara Mpora et al. [63]	Uganda	2014	Cross-sectional	Yes	
Islam et al. [64]	Bangladesh	2017	Cross-sectional	Yes	
Hossain et al. [65]	Malaysia	2013	Cross-sectional	Yes	
Padez [66]	Mozambique	2003	Cross-sectional	Yes	
Ramalingam & Veeraraghavan [67]	India	2019	Cross-sectional	Yes	
Padez [68]	Portugal	2003	Cohort	Yes	
Ali et al. [69]	Sudan	2011	Cross-sectional	Yes	

### The effect of sample size

The sample size can have an impact on public health research [70]. The review revealed that studies with higher samples had a lower mean age at menarche for both rural and urban areas than those with smaller samples, which corroborates an earlier study that reported the same [71]. A larger sample size generally provides a more reliable estimate of the average age of menarche within a population. With a larger sample size, the variability in menarcheal age among individuals is more likely to be represented accurately, which can lead to more robust conclusions about mostly nongenetic factors influencing menarcheal age. On the other hand, in studies with small sample sizes, the average age of menarche may be less representative of the true population average, and the results may be more susceptible to random fluctuations. Additionally, smaller sample sizes can limit the ability to detect significant differences in menarcheal age between subgroups within the population, such as rural and urban areas, and consequently reduce the reliability and generalizability of the resulting findings.

### Regional differences in menarcheal age in Nigeria

Nigeria is divided into two major regions, the northern and the southern part. This review joins a lot of earlier studies that investigated regional differences in public health research in Nigeria. This research has shown that girls in southern Nigeria experienced menarche earlier than those in the North, which corroborated a similar study [72]. However, the authors collected data from previous demographic surveys. Various factors could be responsible for the observed differences. Some of the factors are genetic, ethnic, nutritional, socioeconomic, environmental, and cultural variables. The differences between menarcheal ages of northern and southern Nigeria are not statistically significant, corroborating a study that reported the same for pubarche in girls [73].

### Distribution of rural and urban menarcheal ages

The Gaussian kernel estimated a bimodal distribution for rural girls, where they are most likely to experience menarche at 11 and 13 years, respectively. The bimodal distribution suggests that rural girls experience menarche in two clusters: the early onset (a significant portion of girls reach menarche around 11 years old) and later onset (another group experiences menarche at the more typical age of 13 years old). Urban girls are most likely to experience menarche at 13 years. In both locations, the incidence of menarche decreases just after the peaks at 13 years. The presence of these two peaks in the distribution of the menarcheal ages of girls in rural Nigeria indicates that the timing of menarche in rural girls is not uniform and that there is variability in the age at which girls reach this milestone. Lastly, the heavy tail on the right side of the rural MAM distribution just after the peak at the age of 13 could imply that there is greater variability in the ages, which results in the reported delayed menarche when compared with those living in urban areas.

### Limitation

Three limitations were identified, and efforts were made to mitigate the associated risks. First, since the menarcheal ages were self-reported by the participants in the included articles, questions arose regarding the reliability of the studies. However, studies that reported menarcheal age in menopausal women were excluded to reduce the risk of recall bias. Second, it was unclear whether the adolescents were also schoolgirls. To address this, the authors divided the sample size into adolescents and schoolgirls. Third, the systematic review did not comprehensively cover all the geopolitical regions (GPR) in Nigeria, potentially leading to biases if certain areas with distinct socioeconomic and cultural characteristics were underrepresented.

### Implications for research

There will be a need for a detailed dive into how rural and urban differences in living conditions (housing, sanitation), lifestyle factors (diet, physical activity), access to healthcare, and exposure to environmental pollutants contribute to the declining menarcheal age in Nigeria. The analysis could equally be extended to GPR and regional (north vs. south) disparities in the generational fall in the MAM over the years. The specific differences in living conditions, lifestyle, nutrition, access to healthcare, physical activity levels, and exposure to environmental pollutants between rural and urban areas in Nigeria can significantly impact menarcheal age. Urban areas typically have better access to healthcare facilities, higher nutritional standards, and more opportunities for physical activities through structured programs and facilities [74]. Conversely, rural areas may lack these amenities, leading to delayed onset of menarche [75] and other reproductive health indicators. Additionally, urban areas might have higher exposure to certain environmental pollutants, which can also affect menarcheal age [76], especially those living in the industrialized regions of Lagos and Port-Harcourt, Nigeria.

The northern and southern regions of Nigeria differ significantly in terms of socioeconomic status, education, nutritional status, environment, and culture [77]. The southern region generally has higher socioeconomic status, better access to healthcare, and more urbanized environments, leading to an earlier menarcheal age. In contrast, the northern region may have lower socioeconomic status, limited healthcare access, and more rural settings, contributing to later menarcheal age [78]. Cultural practices and dietary habits also vary, with the north potentially adhering to more traditional lifestyles that can impact nutrition and overall health, thereby affecting the timing of menarche. Research is needed to deepen our understanding of how these regional differences can help in developing targeted public health interventions and policies.

Future research should focus on longitudinal studies to better understand the regional and rural-urban disparities, trends, and causative factors affecting menarcheal age over generations. This will help to capture the factors affecting the receding age of menarche. There is also a need to conduct studies with larger and more representative samples in both rural and urban areas and across northern and southern Nigeria. Also, homogenous populations such as ethnic groups, girls living in difficult terrain, those living with disabilities, or those having sickle cell anemia. Comparative and time-series analyses could be explored.

This review excluded menopausal women to reduce the recall bias. Future research should compare menarcheal ages reported by adolescent girls and menopausal women to determine if there are significant differences between the two groups.

### Policy implication

Policymakers should be proactive in addressing the declining age of menarche, as studies have shown that the earlier onset of menarche is associated with early sexual initiation [79] and other risky sexual behaviors [80], which in turn increases the risk of unwanted pregnancies and sexually transmitted diseases [81, 82]. Hence, compulsory comprehensive sexuality education (CSE) is highly recommended to implement age- and sex-specific and evidence-based CSE in schools to equip adolescent girls with the knowledge and skills to make informed decisions about their bodies and sexual health. Most importantly, efforts should be made to include out-of-school girls in various interventions in this context because out-of-school children engage in high-risk sexual behaviors and are often victims of sexual violence and exploitation [83].

Actions should be taken by policymakers to implement policies and interventions to improve access to nutritious food for adolescent girls in both rural and urban areas. This could involve school feeding programs and supplementation, which could also be supplemented with public health education to disseminate and advocate for healthy lifestyle habits, including but not limited to balanced diets, regular physical activity, and the importance of a healthy weight, to address potential contributing factors to the decline in menarcheal age.

Since it has been reported that primary health care is weak in rural areas of Nigeria [84], targeted actions should be intensified to narrow the rural-urban disparity by removing barriers to and improving access to quality healthcare services in rural areas. This could involve mobile clinics, telemedicine services, and community health worker programs. Also, investment in rural infrastructure is needed to improve water, sanitation, and hygiene (WASH) facilities in rural schools to promote menstrual hygiene management (MHM) [85].

## Conclusion

This systematic review provided evidence on the rural-urban menarcheal differences in Nigerian girls. The average age of menarche is declining across Nigeria, like in other countries. The review found no significant difference between rural and urban areas overall, but girls in southern Nigeria tend to experience menarche earlier than those in the northern region. This finding is consistent with studies from Poland, Cameroon, India, China, and others, indicating a generational difference in menarcheal age between rural and urban areas. Interestingly, rural girls show a bimodal distribution, with peaks at 11 and 13 years old. This suggests two groups: some starting puberty early and others at the more typical age of 13. Urban girls have a single peak at 13. The sample size was also found to impact the estimation of menarcheal age, with larger samples providing more reliable estimates. Overall, this study highlights the variability in menarche timing and the need for further research into the reasons behind regional and rural-urban differences.

## Electronic supplementary material

Below is the link to the electronic supplementary material.

Supplementary Material 1

## Data Availability

The data that support the findings of this study are provided in the Supplementary Materials.

## Abbreviations

GPR: Geopolitical region
KDE: Kernel density estimation
MAM: Mean age at menarche
MHM: Menstrual hygiene management
PICO: Population Intervention Comparator Outcome
PRISMA: Preferred Reporting Items for Systematic Reviews and Meta-Analyses
STI: Sexual transmitted infection
STD: Sexual transmitted disease
WASH: Water, sanitation and hygiene

## References

[1] Zehravi M, Maqbool M, Ara I. Teenage menstrual dysfunction: an overview. Int J Adolesc Med Health. 2023;35(1):15–9. 36117242 10.1515/ijamh-2022-0018

[2] Maqbool R, Maqbool M, Zehravi M, Ara I. Menstrual distress in females of reproductive age: a literature review. Int J Adolesc Med Health. 2022;34(2):11–7. 10.1515/ijamh-2021-0081 34293834

[3] Canelón SP, Boland MR. A systematic literature review of factors affecting the timing of menarche: the potential for climate change to impact women’s health. Int J Environ Res Public Health. 2020;17(5):1703. 32150950 10.3390/ijerph17051703 PMC7084472

[4] Olgun EG, Cetin SK, Siklar Z, Aycan Z, Ozsu E, Ceran A, Berberoglu M. Investigation of early puberty prevalence and time of addition thelarche to pubarche in girls with premature pubarche: two-year follow-up results. Clin Pediatr Endocrinol. 2022;31(1):25–32. 35002065 10.1297/cpe.2021-0042 PMC8713059

[5] Marques P, Madeira T, Gama A. Menstrual cycle among adolescents: girls’ awareness and influence of age at menarche and overweight. Revista Paulista De Pediatria. 2022;40:e2020494. 35019010 10.1590/1984-0462/2022/40/2020494 PMC8734600

[6] Di Sessa A, Grandone A, Marzuillo P, Umano GR, Cirillo G, del Giudice M, E. Early menarche is associated with insulin-resistance and non-alcoholic fatty liver disease in adolescents with obesity. J Pediatr Endocrinol Metab. 2021;34(5):607–12. 33823088 10.1515/jpem-2020-0684

[7] Cheng TS, Day FR, Lakshman R, Ong KK. (2020). Association of puberty timing with type 2 diabetes: a systematic review and meta-analysis. PLoS Med, 17(1), e1003017. 10.1371/journal.pmed.1003017 PMC6944335 31905226

[8] Bubach S, Horta BL, Gonçalves H, Assunção MCF. Early age at menarche and metabolic cardiovascular risk factors: mediation by body composition in adulthood. Sci Rep. 2021;11(1):148. 33420216 10.1038/s41598-020-80496-7 PMC7794383

[9] Yang PJ, Hou MF, Ou-Yang F, Tsai EM, Wang TN. Association of early-onset breast cancer with body mass index, menarche, and menopause in Taiwan. BMC Cancer. 2022;22(1):259. 35277131 10.1186/s12885-022-09361-2 PMC8917681

[10] Yang Y, Wang S, Cong H. Association between age at menarche and bone mineral density in postmenopausal women. J Orthop Surg Res. 2023;18(1):51. 36650576 10.1186/s13018-023-03520-2 PMC9843934

[11] Traggiai C, Stanhope R. Delayed puberty. Best Pract Res Clin Endocrinol Metab. 2002;16(1):139–51. 11987904 10.1053/beem.2001.0186

[12] Leone T, Brown LJ. (2020). Timing and determinants of age at menarche in low-income and middle-income countries. BMJ Global Health, 5(12), e003689. 10.1136/bmjgh-2020-003689 PMC7733094 33298469

[13] e Oliveira KC, Neto JC, Aragon DC, Antonini SR. Nutritional status and age at menarche in amazonian students. Jornal De Pediatria. 2024;100:406–12. 38522477 10.1016/j.jped.2024.03.002 PMC11331225

[14] Dvornyk V. Genetics of age at menarche: a systematic review. Hum Reprod Update. 2012;18(2):198–210. 22258758 10.1093/humupd/dmr050

[15] Euling SY, Selevan SG, Pescovitz OH, Skakkebaek NE. Role of environmental factors in the timing of puberty. Pediatrics. 2008;121(Supplement3):S167–71. 18245510 10.1542/peds.2007-1813C

[16] Osayande SI, Ozoene JO, Amabebe E. Body mass index influences the age at menarche and duration of menstrual cycle. Am J Health Res. 2014;2(5):310–5.

[17] Calthorpe L, Brage S, Ong KK. Systematic review and meta-analysis of the association between childhood physical activity and age at menarche. Acta Paediatr. 2019;108(6):1008–15. 30588652 10.1111/apa.14711 PMC6563453

[18] Okagbue HI, Olawande TI, Odetunmibi OA, Opanuga AA. Mean age of menarche and the probability of attaining menarche for Nigerian girls. JP J Biostatistics. 2022;21:89–102.

[19] Abioye-Kuteyi EA, Ojofeitimi EO, Aina OI, Kio F, Aluko Y, Mosuro O. The influence of socioeconomic and nutritional status on menarche in Nigerian school girls. Nutr Health. 1997;11(3):185–95. 9131701 10.1177/026010609701100304

[20] Berheto TM, Mikitie WK, Argaw A. Urban-rural disparities in the nutritional status of school adolescent girls in the Mizan district, south-western Ethiopia. Rural Remote Health. 2015;15(3):404–5. 26235698

[21] Liu W, Yan X, Li C, Shu Q, Chen M, Cai L, You D. A secular trend in age at menarche in Yunnan Province, China: a multiethnic population study of 1,275,000 women. BMC Public Health. 2021;21:1890. 34666747 10.1186/s12889-021-11951-x PMC8524999

[22] Ayazbekov A, Nurkhasimova R, Khudaibergenova SS, Zhunussov D, Zulpukharov A. Puberty start of girls residing in urban and rural areas of the Turkestan region. Adv Gerontol. 2022;12(1):47–55.

[23] Ma N, Shi D, Dang JJ, Zhong PL, Liu YF, Cai S, Song Y. Secular trends and urban–rural disparities in the median age at menarche among Chinese Han girls from 1985 to 2019. World J Pediatr. 2023;19(12):1162–8. 37093553 10.1007/s12519-023-00723-9

[24] Nowak AC, Nutsch N, Brake TM, Gehrlein LM, Razum O. Associations between postmigration living situation and symptoms of common mental disorders in adult refugees in Europe: updating systematic review from 2015 onwards. BMC Public Health. 2023;23(1):1289. 37407937 10.1186/s12889-023-15931-1 PMC10320886

[25] Adeloye D, Ige JO, Aderemi AV, Adeleye N, Amoo EO, Auta A, Oni G. Estimating the prevalence, hospitalisation and mortality from type 2 diabetes mellitus in Nigeria: a systematic review and meta-analysis. BMJ Open. 2017;7(5):e015424. 28495817 10.1136/bmjopen-2016-015424 PMC5566593

[26] Panic N, Leoncini E, de Belvis G, Ricciardi W, Boccia S. (2013). Evaluation of the endorsement of the preferred reporting items for systematic reviews and meta-analysis (PRISMA) statement on the quality of published systematic review and meta-analyses. PLoS ONE, 8(12), e83138. 10.1371/journal.pone.0083138 PMC3873291 24386151

[27] Tawfik GM, Dila KAS, Mohamed MYF, Tam DNH, Kien ND, Ahmed AM, Huy NT. A step-by-step guide for conducting a systematic review and meta-analysis with simulation data. Trop Med Health. 2019;47:1–9. 31388330 10.1186/s41182-019-0165-6 PMC6670166

[28] Must A, Phillips SM, Naumova EN, Blum M, Harris S, Dawson-Hughes B, Rand WM. Recall of early menstrual history and menarcheal body size: after 30 years, how well do women remember? Am J Epidemiol. 2002;155(7):672–9. 11914195 10.1093/aje/155.7.672

[29] Osayande I, Ogunyemi O, Gwacham-Anisiobi U, Olaniran A, Yaya S, Banke-Thomas A. Prevalence, indications, and complications of caesarean section in health facilities across Nigeria: a systematic review and meta-analysis. Reproductive Health. 2023;20(1):81. 37268951 10.1186/s12978-023-01598-9 PMC10237076

[30] Oduntan SO, Ayeni O, Kale OO. The age of menarche in Nigerian girls. Ann Hum Biol. 1976;3(3):269–74. 962305 10.1080/03014467600001431

[31] Wright EA. Menarche in Plateau State of Nigeria. West Afr J Med. 1990;9(3):204–7. 2271434

[32] Ikaraoha CI, Mbadiwe INC, Igwe CU, Allagua DO, Mezie O, Iwo GTO, Ofori PI. (2005). Menarchial age of secondary school girls in urban and rural areas of Rivers State, Nigeria. Online Journal of Health & Allied Sciences, 2: Art. 4.

[33] Tunau K, Hassan M, Ekele B, Adamu A, Ahmed Y. Age at menarche among school girls in Sokoto, Northern Nigeria. Ann Afr Med. 2012;11(2):103–7. 10.4103/1596-3519.93533. 22406670 10.4103/1596-3519.93533

[34] Ekong I, Udofia E, Johnson O, Ekanem US, Okojie O. Menstrual problems and their prevalence among adolescents in Akwa Ibom. Int J Curr Res Acad Rev. 2015;3(8):96–105.

[35] Ekong IE. Perception of menstruation among Adolescent Secondary School Girls in Akwa Ibom State, Nigeria: an implication for Sexual Health Education for Secondary School Girls. Ulutas Med J. 2015;1(3):74–80.

[36] Fagbamigbe AF, Obiyan MO, Fawole OI. (2018). Parametric survival analysis of menarche onset timing among Nigerian girls. Heliyon, 4(12), e01105. 10.1016/j.heliyon.2018.e01105 PMC6310774 30603722

[37] Anikwe CC, Mamah JE, Okorochukwu BC, Nnadozie UU, Obarezi CH, Ekwedigwe KC. (2020). Age at menarche, menstrual characteristics, and its associated morbidities among secondary school students in Abakaliki, Southeast Nigeria. Heliyon, 6(5), e04018. 10.1016/j.heliyon.2020.e04018 PMC7268279 32518847

[38] Edet OB, Bassey PEM, Esienumoh EE, Ndep AO. An exploratory study of menstruation and menstrual hygiene knowledge among adolescents in urban and rural secondary schools in cross river state, Nigeria. Afr J Biomedical Res. 2020;23(3):321–6.

[39] Ovuakporaye SI, Nwangwa EK, Oji BN, Nwaobuoku SU, Onobrakpeya A. Variations in the age of onset of menarche among inhabitants of rural and urban areas in delta state south-south Nigeria. World J Biology Pharm Health Sci. 2023;14(1):168–75.

[40] Spencer J, Angeles G. Kernel density estimation as a technique for assessing availability of health services in Nicaragua. Health Serv Outcomes Res Method. 2007;7:145–57.

[41] Goon D, Toriola A, Uever J, Wuam S, Toriola O. Growth status and menarcheal age among adolescent schoolgirls in Wannune, Benue State, Nigeria. BMC Pediatr. 2010;10:60–60. 10.1186/1471-2431-10-60. 20723237 10.1186/1471-2431-10-60 PMC2939625

[42] Wahab A, Wilopo SA, Hakimi M, Ismail D. Declining age at menarche in Indonesia: a systematic review and meta-analysis. Int J Adolesc Med Health. 2020;32(6):20180021. 10.1515/ijamh-2018-0021 30256760

[43] Lei, Y., Luo, D., Yan, X., Zhang, J., Hu, P., Ma, J., … Lau, P. W. (2021). The mean age of menarche among Chinese schoolgirls declined by 6 months from 2005 to 2014. Acta Paediatrica, 110(2), 549–555. 10.1111/apa.15441 32573028

[44] Jones LL, Griffiths PL, Norris SA, Pettifor JM, Cameron N. Age at menarche and the evidence for a positive secular trend in urban South Africa. Am J Hum Biology: Official J Hum Biology Association. 2009;21(1):130–2. 10.1002/ajhb.20836 18942702

[45] Meher T, Sahoo H. Secular trend in age at menarche among Indian women. Sci Rep. 2024;14(1):5398. 38443461 10.1038/s41598-024-55657-7 PMC10914750

[46] Herman-Giddens ME. The decline in the age of menarche in the United States: should we be concerned? J Adolesc Health. 2007;40(3):201–3. 17321418 10.1016/j.jadohealth.2006.12.019

[47] Piras, G. N., Bozzola, M., Bianchin, L., Bernasconi, S., Bona, G., Lorenzoni, G.,… Perissinotto, E. (2020). The levelling-off of the secular trend of age at menarche among Italian girls. Heliyon, 6(6), e04222. 10.1016/j.heliyon.2020.e04222 PMC7322252 32613111

[48] Sinai T, Bromberg M, Axelrod R, Shimony T, Stark AH, Keinan-Boker L. Menarche at an earlier age: results from two national surveys of Israeli youth, 2003 and 2016. J Pediatr Adolesc Gynecol. 2020;33(5):459–65. 32339696 10.1016/j.jpag.2020.04.005

[49] Garenne M. Trends in age at menarche and adult height in selected African countries (1950–1980). Ann Hum Biol. 2020;47(1):25–31. 31996030 10.1080/03014460.2020.1716994

[50] Iwase, M., Taniyama, Y., Koyanagi, Y. N., Kasugai, Y., Oze, I., Masuda, N., … Matsuo,K. (2024). A Century of Change: Unraveling the Impact of Socioeconomic/Historical Milestones on Age at Menarche and Other Female Reproductive Factors in Japan. Journal of Epidemiology, 10.2188/jea.JE20230155 PMC11230879 38191181

[51] Mangla M. The decline in the age of onset of Puberty–A source of concern. Curr Women’s Health Reviews. 2024;20(3):96–100.

[52] Naz MSG, Farahmand M, Dashti S, Tehrani FR. (2022). Factors affecting menstrual cycle developmental trajectory in adolescents: a narrative review. Int J Endocrinol Metabolism, 20(1), e120438. 10.5812/ijem.120438 PMC8994833 35432553

[53] Łaska-Mierzejewska T, Milicer H, Piechaczek H. Age at menarche and its secular trend in urban and rural girls in Poland. Ann Hum Biol. 1982;9(3):227–33. 7103404 10.1080/03014468200005721

[54] Ajong AB, Tankala NN, Yakum MN, Azenoi IS, Kenfack B. Knowledge of peri-menarcheal changes and a comparative analysis of the age at menarche among young adolescent schoolgirls in urban and rural Cameroon. BMC Public Health. 2020;20(1):1661. 33148224 10.1186/s12889-020-09787-y PMC7641860

[55] Choudhary S, Khichar S, Dabi D, Parakh M, Dara K P, Parakh P, B Deopa. Urban rural comparison of anthropometry and menarcheal status of adolescent school going girls of Jodhpur, Rajasthan, India. J Clin Diagn Research: JCDR. 2016;10(10):SC08–12. 10.7860/JCDR/2016/21882.8757 PMC5121753 27891415

[56] Ohsawa S, Ji CY, Kasai N. Age at menarche and comparison of the growth and performance of pre-and post‐menarcheal girls in China. Am J Hum Biology: Official J Hum Biology Association. 1997;9(2):205–12. 10.1002/(SICI)1520-6300(1997)9:2<205::AID-AJHB6>3.0.CO;2-Z 28561528

[57] Said-Mohamed, R., Prioreschi, A., Nyati, L. H., van Heerden, A., Munthali, R. J.,Kahn, K., … Norris, S. A. (2018). Rural–urban variations in age at menarche, adult height, leg-length and abdominal adiposity in black South African women in transitioning South Africa. Annals of Human Biology, 45(2), 123–132. 10.1080/03014460.2018.1442497 PMC5964443 29557678

[58] Zegeye DT, Megabiaw B, Mulu A. Age at menarche and the menstrual pattern of secondary school adolescents in northwest Ethiopia. BMC Womens Health. 2009;9:29. 19804623 10.1186/1472-6874-9-29 PMC2763859

[59] Mokha R, Kaur AI, Kaur N. Age at menarche in urban-rural Punjabi Jat Sikh girls. Anthropol. 2006;8(3):207–9.

[60] Song Y, Ma J, Wang HJ, Wang Z, Hu P, Zhang B, Agardh A. Trends of age at menarche and association with body mass index in Chinese school-aged girls, 1985–2010. J Pediatr. 2014;165(6):1172–7. 25241174 10.1016/j.jpeds.2014.08.013

[61] Marván ML, Castillo-López RL, del‐Callejo‐Canal DD, Canal‐Martínez ME, la Núñez‐de A. Secular trends in age at menarche in 20th century Mexico: differences by ethnicity, area of residency, and socioeconomic status. Am J Hum Biology. 2020;32(6):e23404. 10.1002/ajhb.23404 32052905

[62] Zakaria R, Amor H, Baali A. Age at menarche and place of residence (Marrakesh, Morocco). Archives De Pédiatrie. 2019;26(1):30–3. 30554849 10.1016/j.arcped.2018.10.001

[63] Odongkara Mpora B, Piloya T, Awor S, Ngwiri T, Laigong P, Mworozi EA, Hochberg ZE. Age at menarche in relation to nutritional status and critical life events among rural and urban secondary school girls in post-conflict Northern Uganda. BMC Womens Health. 2014;14:66. 24885913 10.1186/1472-6874-14-66 PMC4021025

[64] Islam MS, Hussain MA, Islam S, Mahumud RA, Biswas T, Islam SMS. Age at menarche and its socioeconomic determinants among female students in an urban area in Bangladesh. Sex Reproductive Healthc. 2017;12:88–92. 10.1016/j.srhc.2017.03.008 28477938

[65] Hossain MG, Wee AS, Ashaie M, Kamarul T. Adult anthropometric measures and socio-demographic factors influencing age at menarche of university students in Malaysia. J Biosoc Sci. 2013;45(5):705–17. 23480448 10.1017/S0021932013000060

[66] Padez C. Age at menarche of schoolgirls in Maputo, Mozambique. Ann Hum Biol. 2003;30(4):487–95. 12881146 10.1080/0301446031000111401

[67] Ramalingam L, Veeraraghavan A. A cross-sectional study on the age of onset of menarche in females among rural and urban areas of Kanchipuram district in the past 5 years, since 2014. Natl J Physiol Pharm Pharmacol. 2019;10(1):94–94.

[68] Padez C. Social background and age at menarche in Portuguese university students: a note on the secular changes in Portugal. Am J Hum Biology. 2003;15(3):415–27. 10.1002/ajhb.10159 12704717

[69] Ali AA, Rayis DA, Mamoun M, Adam I. Age at menarche and menstrual cycle pattern among schoolgirls in Kassala in eastern Sudan. J Public Health Epidemiol. 2011;3(3):111–4.

[70] Althubaiti A. Sample size determination: a practical guide for health researchers. J Gen Family Med. 2023;24(2):72–8. 10.1002/jgf2.600 PMC10000262 36909790

[71] Nielsen EM. (2011). Trends in the Age of Menarche. Southern Illinois University Carbondale. Honors Theses. Paper 340.

[72] Garenne M. Age at menarche in Nigerian demographic surveys. J Biosoc Sci. 2021;53(5):745–57. 32912346 10.1017/S0021932020000504

[73] Fagbamigbe AF, Obiyan M, Fawole OI. Gender differentials in the timing and prognostic factors of pubarche in Nigeria. PLoS ONE. 2022;17(11):e0277844. 36409757 10.1371/journal.pone.0277844 PMC9678277

[74] Cadmus EO, Adebusoye LA, Owoaje ET. Rural–urban differences in quality of life and associated factors among community-dwelling older persons in Oyo state, South-Western Nigeria. Qual Quant. 2022;56(3):1327–44.

[75] Sommer M. Menarche: a missing indicator in population health from low-income countries. Public Health Rep. 2013;128(5):399–401. 23997288 10.1177/003335491312800511 PMC3743290

[76] Jung EM, Kim HS, Park H, Ye S, Lee D, Ha EH. Does exposure to PM10 decrease age at menarche? Environ Int. 2018;117:16–21. 29704753 10.1016/j.envint.2018.04.020

[77] Eze TC, Okpala CS, Ogbodo JC. Patterns of inequality in human development across Nigeria’s six geopolitical zones. Developing Ctry Stud. 2014;4(8):97–101.

[78] Onyiriuka AN, Egbagbe EE. Anthropometry and menarcheal status of adolescent Nigerian urban senior secondary school girls. Int J Endocrinol Metabolism. 2013;11(2):71. 10.5812/ijem.8052 PMC3693660 23825976

[79] Osemwenkha AP, Osaikhuwuomwan JA, Chukwudi EO. Age at menarche among secondary school girls in an urban population of Nigeria. Nigerian J Experimental Clin Biosci. 2014;2(2):95–9.

[80] Roman Lay AA, Fujimori E, Simões Duarte L, Borges V, A. L. (2021). Prevalence and correlates of early sexual initiation among Brazilian adolescents. PLoS ONE, 16(12), e0260815. 10.1371/journal.pone.0260815 PMC8670678 34905552

[81] Yu EJ, Choe SA, Yun JW, Son M. Association of early menarche with adolescent health in the setting of rapidly decreasing age at menarche. J Pediatr Adolesc Gynecol. 2020;33(3):264–70. 31874313 10.1016/j.jpag.2019.12.006

[82] Whitworth HS, Baisley KJ, Nnko S, Irani J, Aguirre-Beltran A, Changalucha J, Watson-Jones D. Associations between age of menarche, early sexual debut and high-risk sexual behaviour among urban Tanzanian schoolgirls: a cross-sectional study. Tropical Med Int Health. 2023;28(3):237–46. 10.1111/tmi.13858 36717965

[83] Odeyemi K, Onajole A, Ogunowo B. Sexual behavior and the influencing factors among out of school female adolescents in Mushin market, Lagos, Nigeria. Int J Adolesc Med Health. 2009;21(1):101–10. 19526700 10.1515/ijamh.2009.21.1.101

[84] Amedari MI, Ejidike IC. Improving access, quality and efficiency in health care delivery in Nigeria: a perspective. PAMJ-One Health. 2021;5:3.

[85] Lawan UM, Nafisa WY, Musa AB. Menstruation and menstrual hygiene amongst adolescent schoolgirls in Kano, Northwestern Nigeria. Afr J Reprod Health. 2010;14(3):201–7. 21495614

