# Supplementary material for: Assessment of the differences in Mean Age at Menarche (MAM) among adolescent girls in rural and urban Nigeria: a systematic review
Source: BMC Public Health. 2024 Dec 18;24:3468. doi: 10.1186/s12889-024-21054-y (PMC11657176; doi:10.1186/s12889-024-21054-y)
Supplement: Supplementary file 1 — Supplementary Material 1 [file 12889_2024_21054_MOESM1_ESM.docx]

**Assessment of the Differences in Mean Age at Menarche (MAM) among Adolescent Girls in Rural and Urban Nigeria: A Systematic Review**

Hilary I. Okagbue, Olugbemisola W. Samuel, Emmanuella C. Nzeribe, Sunday N. Nto, Olukayode E. Dahunsi, Muhammad B. Isa, John Etim, Evelyn E. Orya, Sidney Sampson, Alexey V. Yumashev

**SUPPLEMENTARY MATERIAL**

**Table S1:** Data Extracted from the studies

| **Citation** | **Study period** | **Overall MAM** | **Rural MAM** | **Urban MAM** | **Sample size** | **Sample composition** | **Study setting** | **Study design** | **Study summary** | **Significance between urban & rural MAM** |
| --- | --- | --- | --- | --- | --- | --- | --- | --- | --- | --- |
| Oduntan, et al. (1976) |  |  | 14.50±0·09 | 13·70±0·03 | 2357 | Adolescent girls | South West | Cross-sectional | Socioeconomic status affect menarche |  |
| Wright (1990) | 1984 | 13.5±1.33 | 14.0±1.40 | 13·20±1.12 | 664 | School girls | North Central | Cross-sectional | Different in social groups |  |
| Ikaraoha et al. (2005) |  |  | 14.22 ± 1.47 | 13.19±1.32 | 859 | School girls | South South | Cross-sectional | Socioeconomic status affect menarche; sporting activities affect menarche | Yes |
| Tunau et al. (2012) | 2007-2009 | 15.26 | 15.32 | 15.2 | 708 | School girls | North West | Cross-sectional | Socioeconomic status affect menarche | No |
| Ekong et al. (2015) |  |  | 13 | 12.7 | 601 | School girls | South South | Cross-sectional | Menstrual problems affects both those in rural and urban | Yes |
| Ekong (2015) |  |  | 12.6±1.8 | 11.8±1.5 | 600 | School girls | South South | Cross-sectional | Difference in knowledge of menstruation |  |
| Fagbamigbe et al. (2018) |  | 13·536 ± 1.789 | 13.47 | 13.59 | 1097 | Adolescent girls | All | Population Cross-sectional | Ethnic differences | No |
| Anikwe et al. (2020) | 2015 | 13 ± 1.0 | 13.3 | 13 | 450 | School girls | South East | Cross-sectional | Socioeconomic status affect menarche | No |
| Edet et al. (2020) |  | 10.95±4.10 | 10.71±0.239 | 11.1±0.157 | 1006 | School girls | South South | Cross-sectional | Difference in knowledge of menstruation |  |
| Ovuakporaye et al. (2023) |  | 12.68 ± 1.365 | 13.26 ± 1.223 | 12.18 ± 1.287 | 243 | School girls | South South | Cross-sectional | Socioeconomic status affect menarche | Yes |

**Table S2:** Risk Assessment Results

| **References** | **S/N** | **Were the criteria for inclusion in the sample clearly defined?** | **Were the study subjects and the setting described in detail?** | **Were objective, standard criteria used for measurement of the condition?** | **Were the outcomes measured in valid and relaible way?** | **Was appropraite statistical analysis used?** | **Rating** | **Comment** |
| --- | --- | --- | --- | --- | --- | --- | --- | --- |
| **[30]** | Oduntan, et al. (1976) | 0 | 0 | 1 | 1 | 1 | 60% | Moderate |
| **[31]** | Wright (1990) | 0 | 0 | 1 | 1 | 1 | 60% | Moderate |
| **[32]** | Ikaraoha et al. (2005) | 1 | 1 | 1 | 1 | 1 | 100% | Very Good |
| **[33]** | Tunau et al (2012). | 1 | 1 | 1 | 1 | 1 | 100% | Very Good |
| **[34]** | Ekong et al (2015). | 1 | 1 | 1 | 1 | 1 | 100% | Very Good |
| **[35]** | Ekong (2015) | 1 | 1 | 1 | 1 | 1 | 100% | Very Good |
| **[36]** | Fagbamigbe, (2018) | 1 | 1 | 1 | 1 | 1 | 100% | Very Good |
| **[37]** | Anikwe et al (2020). | 0 | 0 | 1 | 1 | 1 | 60% | Moderate |
| **[38]** | Edet et al (2020). | 1 | 1 | 1 | 1 | 1 | 100% | Very Good |
| **[39]** | Ovuakporaye et al(2023) | 1 | 1 | 1 | 1 | 1 | 100% | Very Good |
